# Separating mouse malignant cell line (EL4) from neonate spermatogonial stem cells utilizing microfluidic device in vitro

**DOI:** 10.1186/s13287-020-01671-1

**Published:** 2020-05-24

**Authors:** Behnaz Ashtari, Azar Shams, Narges Esmaeilzadeh, Sara Tanbakooei, Morteza Koruji, Mojtaba Johari Moghadam, Javad Mohajer Ansari, Adel Johari Moghadam, Ronak Shabani

**Affiliations:** 1 Shahdad Ronak Commercialization Company, Pasdaran Street, Tehran, Iran; 2 Radiation Biology Research Center, Iran University of Medical Sciences, Tehran, Iran; 3 Department of Medical Nanotechnology, Faculty of Advanced Technologies in Medicine, Iran University of Medical Sciences, Tehran, Iran; 4 Cellular and Molecular Research Center, Iran University of Medical Sciences, Tehran, Iran; 5 School of Mechanical Engineering, Iran University of Science & Technology, Tehran, Iran; 6 Department of Cardiology, Aja University of Medical Sciences, Tehran, Iran; 7 School of Mechanical and Manufacturing Engineering, University of New South Wales, Sydney, New South Wales 2052 Australia

**Keywords:** Microfluidic device, Spermatogonial stem cells, EL4 cancer cell, Cell sorting, Purification

## Abstract

**Background:**

Some children who have survived cancer will be azoospermic in the future. Performing isolation and purification procedures for spermatogonial stem cells (SSC) is very critical. In this regard, performing the process of decontamination of cancerous cells is the initial step. The major objective of the present study is to separate the malignant EL4 cell line in mice and spermatogonial stem cells in vitro.

**Methods:**

The spermatogonial stem cells of sixty neonatal mice were isolated, and the procedure of co-culturing was carried out by EL4 which were classified into 2 major groups: (1) the control group (co-culture in a growth medium) and (2) the group of co-cultured cells which were separated using the microfluidic device. The percentage of cells was assessed using flow cytometry technique and common laboratory technique of immunocytochemistry and finally was confirmed through the laboratory technique of reverse transcription-polymerase chain reaction (RT-PCR).

**Results:**

The actual percentage of EL4 and SSC after isolation was collected at two outlets: the outputs for the smaller outlet were 0.12% for SSC and 42.14% for EL4, while in the larger outlet, the outputs were 80.38% for SSC and 0.32% for EL4; in the control group, the percentages of cells were 21.44% for SSC and 23.28% for EL4 (based on *t* test (*p* ≤ 0.05)).

**Conclusions:**

The present study demonstrates that the use of the microfluidic device is effective in separating cancer cells from spermatogonial stem cells.

## Background

The spermatogonium that are present in the testicles from birth are the precursors to the production of male sex cells. The presence of these kinds of cells is very essential for performing the process of spermatogenesis. But unfortunately, these cells are not capable of making the mature sperm before the puberty period due to the fact that they are dependent on hormonal stimuli [1]. Sometimes, this system is in difficulty, and it is possible to maintain the reproductive capacity and fertility system in men that can ejaculate. In recent years, the success rate of chemotherapy or radiotherapy for the treatment of childhood cancers has been high, whereas more than 70% of children with cancer reach adulthood and reproductive years. Infertility is one of the most common complications of childhood cancer in treated boys in the long run. Unfortunately, about one third of children during their puberty period would experience a considerable decrease in the number of sperms or may face with the medical condition of azoospermia [2, 3]. This can jeopardize their quality of life [4].

In recent years, many attempts have been made to cryopreserve testicular tissue and then to transplant it after chemotherapy and cancer relief, and the willingness of physicians has increased in this way [5, 6]. Cryopreserved testicular tissues have been obtained from boys with cancer before chemotherapy begins to be used to produce sperm germ cells using different culture methods. Before starting chemotherapy, the germ cell (spermatogonial stem cell) is isolated and maintained, and after the patient’s treatment period and after puberty, the cells can be transplanted to the patient and, as a result, fertility can be maintained in these individuals. However, there is a risk of contaminated germ cells taken by tumor cells [7]. The risk of interstitial and intravascular infiltration of testicular tissue among children would increase due to the hematological metastatic spread of childhood solid tumors. Additionally, in children with acute lymphoblastic leukemia (ALL) cancer, the risk of interstitial and intravascular infiltration of testicular tissue is very substantial too. Anyway, among one fifth of patients diagnosed with this kind of tumor, microscopic infiltration of leukemic cells would be seen in their blood tests [8].

Jahnukainen reported that germ cell transplantation from leukemic mice induced tumor formation. Germ cells must, therefore, be completely separated from the tumor cells [7]. Fujita et al. isolated the germ cell from 5 different human cancer cell lines using the FACS technique. In this study, human germ cells from 5 cell lines of leukemia by using anti-MHC-I and CD45+ antibodies (specific tumor cell markers) were isolated. In this study, it is shown that, with this method, spermatogonial transplantation is not safe enough [9]. Geens et al. investigated the separation of germ cells from tumor cells in mice and in human testis cell suspensions using MACS and FACS. Initially, they created a cancerous model in the mouse by infusion of the EL4 tumor cell line; after co-culture isolation, separation of cell suspension was performed using MACS and FACS, and after that, unfortunately one of the 20 transplanted mice got malignant after transplantation. Moreover, Hou et al. [10] have tested the MACS method and showed that the separation method of magnetic-activated cell sorting was not able to remove malignant contamination effectively [10]. As a result, MACS and FACS were not sufficiently effective for complete tumor cell removal from the testicular tissue [11]. Furthermore, these methods are complex and costly, and the survival of cells is poorly reported. Differential plating was also suggested by some articles to enrich spermatogonia in cell suspension derived from the testis sample after enzymatic digestion [12, 13]. Moreover, during this stage of studies performing the identification process of specific markers, isolation and enrichment of undifferentiated spermatogonia from differentiated germ cells and vegetal cells were performed precisely by researchers. However, conducting the process of isolation of blood-related malignancies from germ cells requires a more precise assessment before performing any related clinical procedures. In a study conducted by Dirami and colleagues [12], by utilizing differential adhesion and sedimentation velocity (separating based on shape and size), an isolate of cells was created that contains 95 to 98% porcine type A spermatogonia. In their study, Shinohara et al. [14] demonstrated that by using the technique of laminin adhesion, the process of isolation of spermatogonial stem cells (SSCs) was improved to 3-fold [14]. In another similar study, Morena et al. [15] demonstrated that attaining an 85% isolate of type A, c-kit-positive spermatogonia utilized the technique of sedimentation velocity (SV-AUC) in conjunction with differential adhesion hypothesis (DAH) [15].

Due to the lack of specific SSC cell markers, it should focus on other methods. But it turns out that the focus on destroying cancer cells instead of focusing on healthy cells is also a new method used in recent years. Tumor cells should be targeted as well, and studies have been carried out in this regard.

Shabani et al. demonstrated the effect of chemotherapy with cisplatin on the survival rate of cancer cell lines in acute lymphoblastic leukemia (EL4) and mouse spermatogonial stem cells in vitro. In this study, there were four groups which received various cisplatin dosage (0.5, 5, 10, and 15 mg/ml) and five groups treated as the control group (just received medium). The cells’ viability was examined by colorimetric assay of MTT. Based on their achieved results, the number of both EL4 and SSC with a dose of 15 mg cisplatin decreased significantly compared with the control group (*p* ≤ 0.05). Besides, at different times, there was a significant difference between the half maximal inhibitory concentration (IC50) in doses 10 and 15 mg/ml [16]. Chemotherapy drug release method requires an intelligent tool to select and target cancer cells which today uses nanomaterials to achieve this goal.

In a study conducted by Shabani et al., the anticancer effect of cisplatin encapsulated in spermatogonial stem cells (SSCs) from in vitro and folic acid-conjugated poly(lactic-co-glycolic acid) (PLGA) nanoparticles (NPs) on malignant EL4 cell line of mice was assessed. As their main study outcomes, the rate of caspase 3 and BAX genes in EL4 cells increased, and an increase was observed in the TUNEL-positive cells. Cells treated with carrier nanoparticles were then grafted to the mouse, and no tumor symptoms were observed [17]. Eslahi et al. devised a new method for removing cancer cells through gold nanoparticles (AuNPs) by Folate-Silica-Gold Nanorods (F-Si-GNRs); based on their outcomes, in comparison with SSCs, an increment in the signs of F-Si-GNR toxicity was observed in EL4 cells [18]. On the other hand, as shown by Beebe et al. [19], conducting the sorting process of microfluidic cell may be one of the most critical techniques for isolating the immature cells of spermatogonia based on their density and size [19].

The advancement of microfluidic technology has had a tremendous impact on the progress of cell biology science [20]. The benefits of this new method to non-traditional and traditional methods include controlling 3-D culture conditions, having micro-scale physical and fluid properties, and creating multiplexed nanoliter arrays and paths to improve biological research [21–23].

**Fig. 1:**
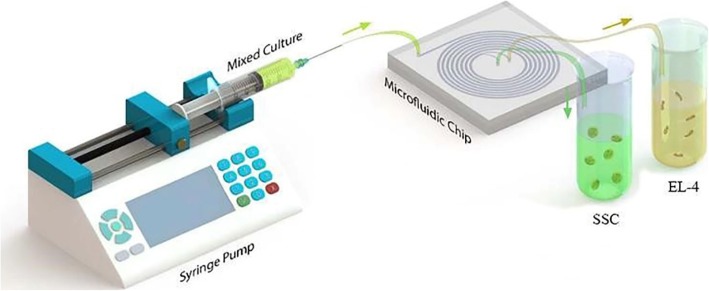
A schematic image of a microfluid [23]

Very low sample size, very fast processing, multi-functionality, and a very large volume/volume ratio of microfluidic system are features [24, 25] that offer new opportunities for cytology and cytopathology, especially for cell sorting and detection [26–32]. Leveraging these advantages, various microfluidic platforms have been developed (Fig. 1). The next important step is to expand microfluidic systems for greater and more efficient use, commercialization and ease of use, industrial improvements, and more effective cost reductions for a long-term continuous perfusion cell culture like a bioreactor [33].

In this current study, we presented a new technique of cell sorting of spermatogonial stem cells from cancerous cells. Based on the performed studies, the present study is the first scientific trial that has focused on research investigating the process of delivering a microfluidic-based cell culture system for achieving this objective. For spreading the advantages of applying microfluidic technology to a broader practical scope, the aforementioned methodology must be developed and integrated into research and screening laboratories.

## Methods

### Device designed and fabricated

With respect to the advent of the novel technique of separation, a new spiral microfluidic device was developed by Warkiani et al. [34]. The pattern of the designed spiral chip device (in the CAD software environment) with an eight-loop spiral microchannel has a unique inlet and two separate outlets with a variable radius that varies from 8 to 24 mm. The cross section (channel width) is 600 μm, and the heights of the inner and outer sections were fitted at 80 and 130 μm respectively as the best values for the trapezoid cross section. Then, to create a master, the pattern was printed by a high-resolution printer and was used as a mask in photolithography on a SU-8 (MicroChem) spin-coated on a on a thin slice of semiconductor (silicon wafer). The device was prepared by mixing of PDMS prepolymer and the curing agent (10:1 ratio) (sylgard 184 Dow Corning) and by degassing onto the master and backing for 2 h at 70 °C. After the baking procedure, the replica of PDMS was peeled off from the master. The surface of the two PDMS replica was oxidized by plasma machine and was bonded irreversibly together. To enhance the bonding, the device was placed inside an oven (30 min at 70 °C). Both of fluidic inlets and outlets were punched (with 1.5 mm diameter) and connected respectively to the syringe pump and the output container (two sterile 15 mL falcon) by Tygon® tubing.

### Sample processing and cell loading

Before sample processing, the spiral chip was washed by ethanol 70% and sterile medium. Using a syringe driver, all samples were split through the inlet into the spiral microfluidic device. For performing the cell separation process appropriately, the optimized flow rate of the sample in microchannels was 1.7 ml/min; typically, into the spiral microchannel inner wall flows the cells larger than 12 μm and cells smaller than that flow into the outer wall. In the end, these cells will separate by inner and outer outlets. The collected cells can then be analyzed by suitable downstream techniques such as immunostaining.

### Isolation of mouse spermatogonial stem cells

For conducting the present examination, sixty neonatal mice models in the age range of 3 and 6 days old were chosen. All of these mice were taken from the National Medical Research Institute (Tehran, Iran). They were kept in cages made of plastic in a room at a temperature range of 22–25 °C, with a 12-h light/dark cycle. The mice could freely reach drinking water and standard laboratory pellets. All animal experiments were approved by the Animal Ethics Committee at Iran University of Medical Sciences (code: IR-IUMS.95-04-117-29910). Nearly all germ cells of testicles were isolated by means of the aforementioned techniques along with some modifications [16, 35]. Mouse testes were collected in phosphate-buffered saline (PBS, Invitrogen, USA) and penicillin/streptomycin. After decapsulation, we used a 2-step enzymatic digestion protocol to obtain a single cell suspension. The testes were mechanically dissociated by two-step enzymatic digestion. The testicles were mechanically and enzymatically digested and isolated. In the first stage, the testicles were divided into smaller pieces and incubated in enzymatic solutions. The first stage enzyme solution contains the following: Dulbecco’s modified Eagle’s medium (DMEM/f12) with 0.05 mg/ml DNase, 1 mg/ml trypsin, and 1 mg/ml collagenase for about half hour with pipetting and shaking at 37 °C for a period of 15–30 min. The digestion process of tissue was carried out by enzyme washing and then centrifugation, and finally, through draining the supernatant solution, the interstitial cells were removed from the seminiferous tubules. The remnants of non-digested seminal tubes entered the second stage of enzymatic digestion so that all cells were extracted from the tubes at this stage. Finally, the isolated spermatogonium and Sertoli cells were cultured in special culture media in DMEM/f12 medium (DMEM/f12; Gibco, Paisley, UK), non-essential amino acids, 2% Bovine serum albumine (BSA) (Sigma, MO, USA), 100 μg/ml streptomycin, and 100 IU/ml penicillin (from Gibco, Paisley, UK). It was a conventional cell condition, and it was continued for 2 weeks to increase the cell number. Finally, in the present study, three main groups were designed, and by accessing the culture collection of Pasteur Institute, Tehran, Iran, the mouse acute lymphoblastic leukemia cell line EL4 was prepared.

### Identity confirmation of the spermatogonial cell by RT-PCR

The confirmation of the nature of spermatogonial cells in the culture medium was investigated by expressing specific genes of these cells according to previous studies.

Testicular cells before cultivation and total RNA molecules as a positive control obtained from the testis samples were extracted by the standard extraction RNX-plus kit in accordance with guidelines presented by its manufacturer (Cinnagen, Iran). Then, the integrity and purity RNA was examined using a ratio measurement of 26/28 nm. Aimed to eliminate residual genomic DNA (gDNA) contamination, total RNA was treated by means of deoxyribonuclease I (DNase I). Initially, by means of SuperScript II Reverse Transcriptase (RT) system and Oligo (dT)_18_ Primers, strand complementary DNA (cDNA) synthesis was performed.

Human integrin α6 (IGα6) and GDNF family receptor alpha-1, known as specific primers of promyelocytic leukemia zinc finger (PLZF) protein, were designed by means of human sequences that were described before (Cinnagen). Aimed to control normalization of polymerase chain reaction (PCR), the β-actin gene was included as a housekeeping gene. Under a specific condition, the laboratory technique of reverse transcription-polymerase chain reaction (RT-PCR) was carried out by means of precisely prepared PCR SuperMix (Cinnagen), primers, and cDNA. The specific provided conditions include the following: 35 cycles at 95 °C for a period of 30 s; during a period of 45 s, for each primer, a specific annealing temperature was prepared (β-actin, 60 °C; Igα6 and GFRα1, 58–62°C; and PLZF, 55 °C); and finally at the temperature of 72 °C for a period of 45 s. For dividing the products of polymerase chain reaction (PCR), approximately 1 μl of each prepared sample was resolved in an agarose gel of 1.7% for electrophoresis. Then, the electrokinetic process of electrophoresis was performed with the help of a working solution of 1× TAE buffer and a voltage of a 95 W for a period of 45 s (Fig. 3).

### Cell confirmation by flow cytometry

For confirmation of the cell value, spermatogonial stem cells (SSCs) were fixed in a paraformaldehyde (PFA) product of 4% in the buffer solution of phosphate-buffered saline (PBS) with a pH of 7.4. After that, the process of washing of cells was conducted three times with paraformaldehyde (PFA) product, incubating in a 1% nonionic surfactant of Triton X-100 in phosphate-buffered saline for a period of half hour and blocking in 0.5% liquid of Bovine serum albumine (BSA) and finally in PBS buffer solution for about half hour. Then, the cells were incubated in a special antibody solution that contains a primary antibody protein of PLZF (Abcam) at 4 °C for a period of time less than 45 min and were then examined. The malignant cell line of EL4 was fixed in a paraformaldehyde (PFA) product of 4% in a PBS buffer solution with a pH of 7.4 and then for a period of half hour washed with the buffer solution of PBS and was incubated with a special H-2Kb antibody (Abcam) and finally assessed precisely (Fig. 5).

### Tumorigenic evaluation of cells after microfluidic isolation

For this purpose, we first transformed healthy rats into azoospermia mice by intraperitoneal injection of busulfan 4 weeks before transplantation. Then, for tumor evaluation, cells were transplanted into azoospermia mice after microfluidic gates, and after 8 weeks, the tumor status was checked (Fig. 7).

### Statistical analysis

The procedure of analyzing data was performed using SPSS Statistics V22, and the statistical significance threshold was determined to be *p* ≤ 0.05. In this study, the Tukey test and an independent *t* test were used for comparing the cell percentages.

## Results

**Fig. 2:**
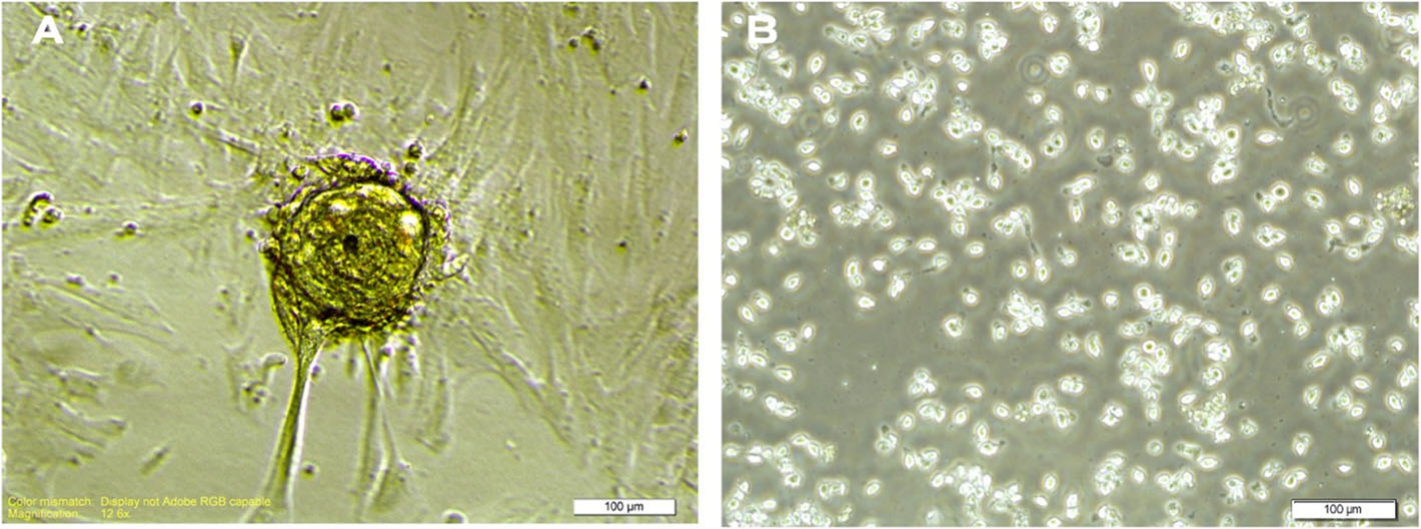
SSC colonies of mouse neonate spermatogonial stem cell after 2 weeks of culture in free-growth factors DMEM/F12, 1 week after primary culture, and EL4 tumor cell. **a** Complete colony of spermatogonium cells. **b** Tumor cells. Scale bars = 100 μm

EL4 tumor cell culture was performed in DMEM/F12 medium with FBS 2%, and the percentage of viable cells was about 80 ± 2.4%. The nature of this cell line during the culture was suspended cells and did not stick to the culture dish. As shown in Fig. 1, their appearance was not spherical and did not form a colony. After 24 h, there was a significant amount of cell clinging to the flask. By invert microscope examination, spermatogonium was circular or oval, with a large nucleus and a small cytoplasm. The isolated SSCs tend to form colonies and form a small cell cluster. The proliferation rate of these cells was very high, with almost every 48 h of cell passage. Also, El4 cells, which were suspended in culture medium, were simultaneously cultured and stored (Fig. 2).

In order to proliferate spermatogonial stem cells, these cells were cultured in DMEM/F12 medium including 2% FBS with GDNF 20 ng/ml and 10 ng/ml BFGF for 2 weeks. At the end of the first week, the process of formation of cluster stem cell assemblages started after about 4 h since in the first passage and a large number of stem cells were colonized in a colony (Fig. 2) culture medium.

### Expression of specific genes of SSCs and EL4 cells using RT-PCR

**Fig. 3:**
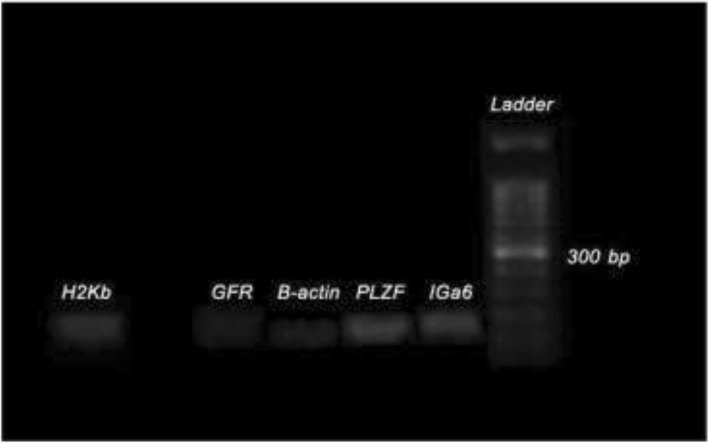
Results of RT-PCR production of spermatogonial stem cells and EL4 cells. The expression of PLZF, GFRα-1, and Integα-6 in spermatogonial stem cells and H2Kb for EL4 cells. β-actin was included as a housekeeping gene

As could be seen from Fig. 3, specific markers of spermatogonial stem cells (SSCs) (Integα-6, GFRα-1, PLZF) in cells after 2 weeks of culture (SC2) and the EL4 marker of H2K-b EL4 cells from product excretion RT-PCR have been proven. Also, β-actin was also observed as the house keeping gene in both samples.

### Determination of the percentage of EL4s and SSCs after microfluidic separation by flow cytometry

**Fig. 4:**
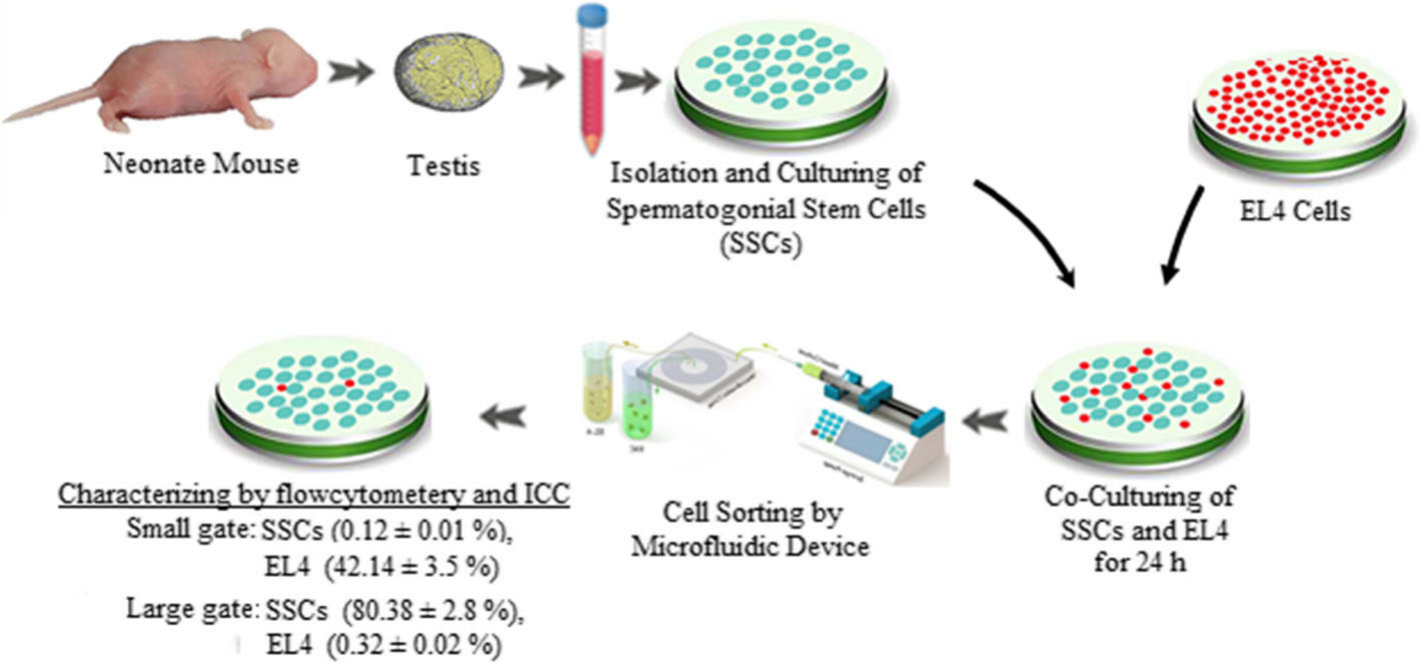
A schematic illustration of how microfluidics works. In this study, adequate separation was performed after transferring the cells to the corresponding device

**Fig. 5:**
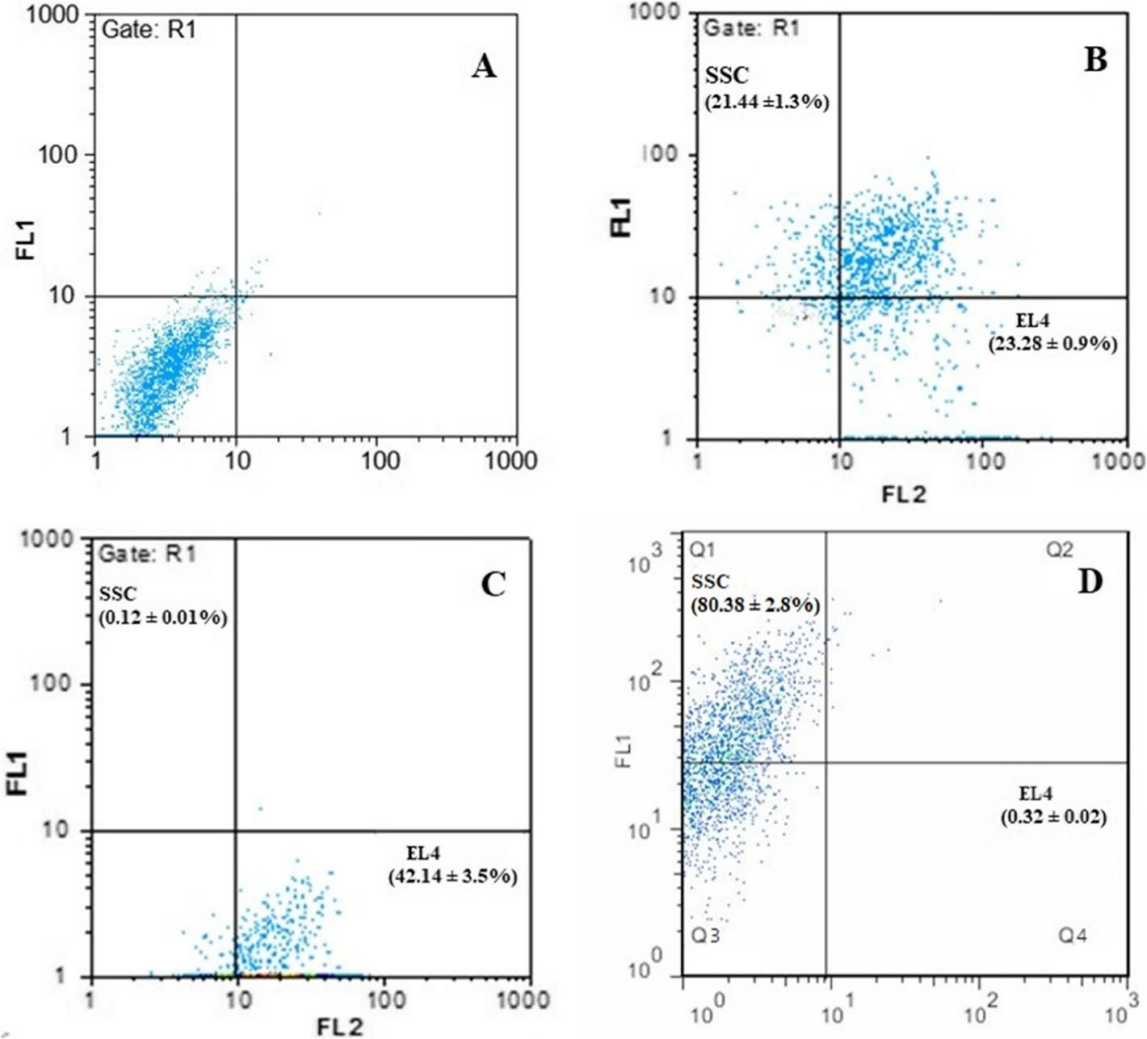
Results of flow cytometry in the mixture of cancer cells and spermatogonial stem cells after microfluidic isolation. **a** The results obtained from cells without adding antibodies. **b** The results of EL4 cell composition and SSCs (in vitro tumor model). **c** The results of cell percentage after passing microfluidic of outer outlet. **d** The results of the percentage of cells associated with the inner outlet. Q1, the range of PLZF positive cells and negative H2k cells; Q2, the range of PLZF positive cells and positive H2k cells, Q3, the range of negative PLZF cells and negative H2k cells; Q4, the range for negative PLZF and positive H2k

In order to evaluate the percentage of spermatogonial stem cells and tumor cells, flow cytometry was used to determine the percentage of the cells. As shown in Fig. 3, the percentage of tumor cells and spermatogonial stem cells after microfluidic isolation was collected at two outlets, and the outer outlet were approximately 0.12 ± 0.01% (SSC) and 42.14 ± 3.5% (EL4). While the outputs collected from the device for inner outlet were 80.38 ± 2.8% (SSC) and 0.32 ± 0.02 (EL4) in the control group, the percentages of SSC and EL4 cells were 21.44 ± 1.3% and 23.28 ± 0.9%, respectively, which did not enter the microfluidic apparatus and were individually mixed in a cell dish (based on *t* test (*p* ≤ 0.05) (Figs. 4 and 5).

### Immunocytochemistry

**Fig. 6:**
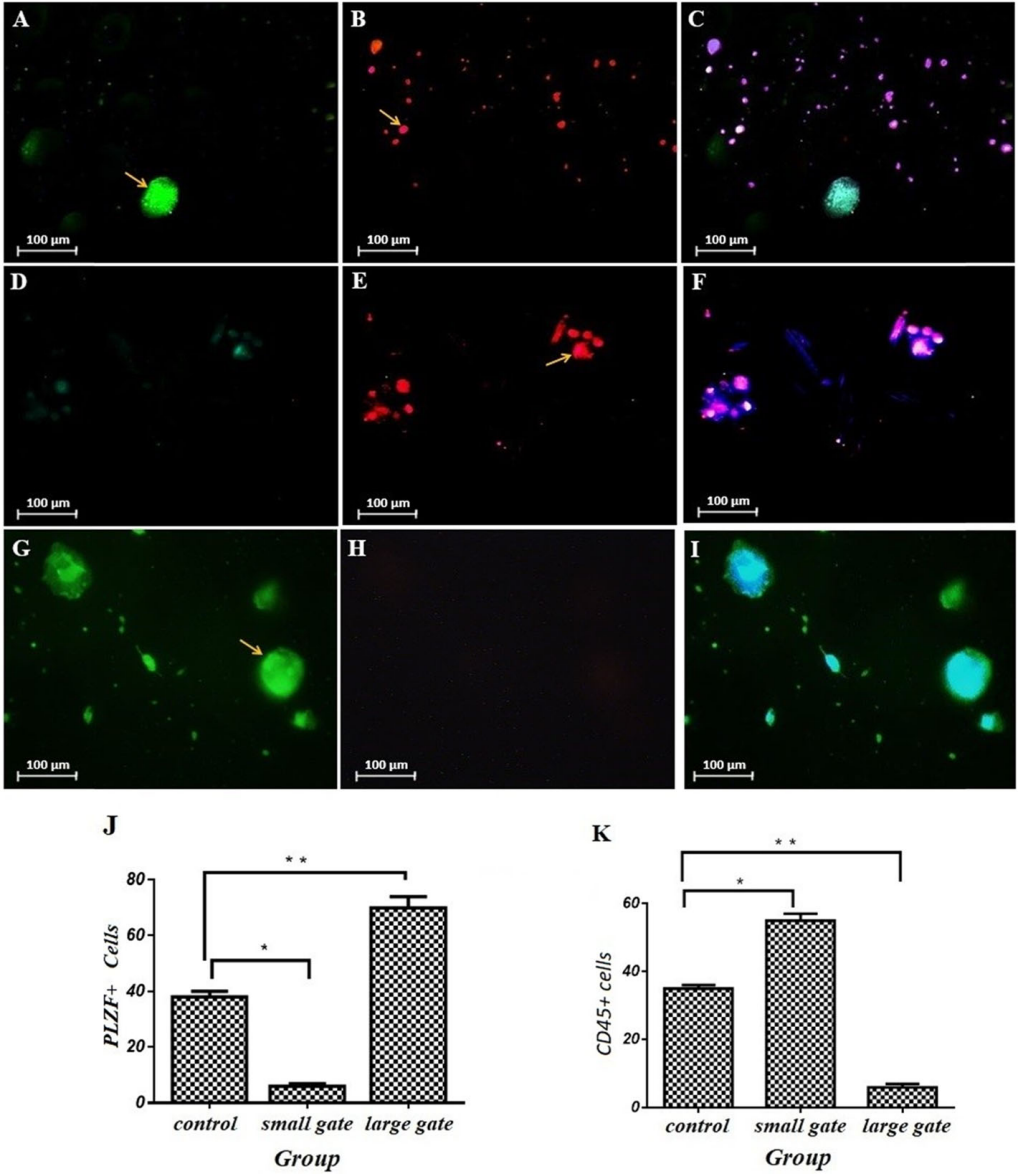
Part A is for the PLZF marker for SSC cells and part B is for tumor cell CD45 markers and the MERGE-shaped image of the above images and DAPI staining. Based on flow cytometry, in the first row, the control group, which contains the SSC and EL4 cell composition, is seen, and both types of cells are seen. In the 2nd row is the output from the outer inlet, which indicates a higher number of EL4 cell. The 3rd row refers to the output of the inner outlet which indicates a higher number of PLZF markers, the SSC cell

After isolating the SSC and EL4 cellular composition using the microfluidic device, the cells were cultured in separate plates for 1 week, and after the end of the first week, the immunocytochemistry test was performed to confirm the microfluidic cells, so for cell EL4, the conjugated CD45 marker was PE, and for the SSCs, the PLZF conjugate marker was used with FITC, which was initially fixed at 4 °C in PBS with PH7.4 for 20 min. After three times washing with PBS, the cells were exposed to Triton X-100 for 10 min in order to penetrate the cells, and then, after three times, the PBS was incubated in 10% goat serum (Sigma, Missouri, USA) for 1 h. It was then incubated with 10 μg/ml antibodies for CD45 and PLZF for 2 h at room temperature. Then, it was washed with 1% goat serum in PBS three times and incubated with FITC-conjugated secondary antibody for 2 h at RT away from light, PE-conjugated, and stained with PBS for coloration of the cells with DAPI for 3 min. The coverslips were mounted and observed under a Nikon Eclipse TE300 Inverted microscope (country). Sample images were captured by the CCD camera directly connected to the microscope mentioned above (Fig. 6). The ICC data were quantified on the basis of ImageJ software and were analyzed by one-way ANOVA, and the results showed that for the smaller gate output, the number of CD45+ was more than the number of PLZF+ cells and was statistically significant (55 ± 2% CD45+ vs 6 ± 1% PLZF+, control 35 ± 1%, *p* ≤ 0.005), and for the larger gate output, the number of PLZF+ cells was statistically significant than CD45+ (70 ± 4% PLZF+ vs 6 ± 1% CD45+, control 38 ± 2%, *p* ≤ 0.005).

### Histological assessment

**Fig. 7:**
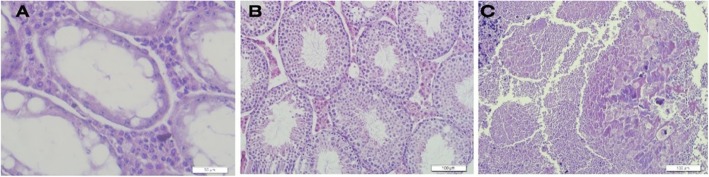
Tissue section confirmation of tumor model and cellular transplantation after microfluidic isolation. **a** Histology section of the testicular tissue in the control group (azoospermia-busulfan model). Scale bar = 50 μm. Empty spaces in the basal part of the seminal tubes indicate the removal of germ cells. **b** Seminal tubes in a group consisting of spermatogonial stem cells after microfluidic isolation. Scale bar = 100 μm. **c** Histopathologic section of the testicular tumor. Scale bar = 100 μm. Pathological changes in testicular tissue are seen (H & E)

The tissue sections of testis after transplantation were checked to confirm the tumorigenicity of the cells and pathological changes from the tumor. As shown in Fig. 7c, compared with the control group (a), the arrangement of epithelial cells of the seminal tubes in the tumor model was degraded and uncertain so that the structure of tissue and tubes was severely disrupted. Other typical pathological changes associated with testicular cancer, including tumor invasion and hyperplasia of the testicular tissue, high numbers of lymphocytes, and the loss of the order of the spermatozoa tubes, were seen. In histopathologic sections of the EL4 cell line and SSCs, no pathologic evidence of tumor was detected after microfluidic isolation, and seminal tubes with normal appearance in histological sections were observed. In addition, spermatogonial stem cell transplantation has improved the relative position and structure of seminal tubes and the onset of spermatogenesis in many seminal tubes (b).

## Discussion

Over the recent decades, significant advances have been made in cancer diagnosis and treatment and have led to an increase in the survival of children. Hence, in the present study, one of the most critical challenges in the preservation of male fertility is highlighted which is the destructive effect of chemotherapy on reproductive function among men, especially in the process of spermatogenesis. At the present time, one of the most critical ways for preserving the natural capability of fertility among boys before their puberty period is performing the process of testicular tissue cryopreservation. This procedure is performed mainly for future isolation and transplantation of spermatogonial stem cells for restoring the process of spermatogenesis [36].

The main concern in the cryopreserved testicular tissue is the potential of cancer cells in the biopsy. Patients with non-tumor cancers are more likely to be at risk, the resumption of cancer [36]. In addition to the available risks of reintroducing lingering tumor cells, the culture system of SSCs could provide the opportunity of selecting the best way for the isolation of cancer cells from healthy cells.

The high precision and significant amount of cell separation should be the main feature of cell separation methods. As long as traditional methods can have high efficiency in the process of cell sorting in a short period of time, the achieved progress in microfluidics has reinforced the realization of miniaturized devices to be able to offer similar capabilities that could extract a broad range of physical principles.

Cell separation methods are rapidly expanding to allow them to target and isolate small numbers of cells such as circulating fetal cells (CFCs), hematopoietic stem cells (HSCs), and circulating tumor cells (CTCs) from the blood [37–39]. Several methods for cell sorting are currently underway, with a large and clear limitations that include the following: low sampling rate and small samplers that do not have the ability to work on larger samples at a wider scale and in a shorter period of time (more than 500 million cells), high operating pressures that can reduce the viability and/or function of equipment and devices that occupy a lot of space, and enough experience to work with the above equipment and increased risk of safety concerns and sample contamination because of performing an aerosolized sample sorting procedure [40]. Older methods for isolating cells have disadvantages, for example, in FACS, the disadvantage for sorting cells is electrolysis of water based on the current of electricity between the cathode and anode poles, which could be one of the main causes of generating harmful compounds like hydrogen peroxide (H_2_O_2_) and bubbles, which would affect cell viability and survival and also the pH of a solution seriously if not be regulated and monitored appropriately, or the use of nanoparticles to remove cancer cells and isolate healthy cells, which are considered as newer methods [41, 42]. Studies have shown that nanoparticles and exposure to them can have devastating effects on prokaryotic cells and complex eukaryotes such as humans. Many studies have shown that nanoparticles can cause degradation of DNA, inflammatory response, oxidative stress, lipid peroxidation, apoptosis, carcinogenicity, non-genotoxic (NGTX), immunotoxicity, alterations in gene expression, reproductive toxicity, cytotoxicity, and genotoxicity [43–50]. The epigenetic changes are among the main available mechanisms for genotoxicity and toxicity of nanoparticles that happen in the specific DNA methylation patterns that may cause alternations in the process of gene expression [51]. On the other hand, although proteomic and genomic data suggest changes in the profile of protein and gene of cells exposed to NP, epigenetic changes are underestimated [49, 52]. Due to the fact that microchips could precisely control temporal and spatial conditions in an appropriate miniaturized droplet microarray platform, they could easily monitor and control cells [20, 53].

In this study, the percentages of tumor cells and spermatogonial stem cells after microfluidic isolation were collected at two outlets, and the outputs for smaller tumor cells were approximately 0.12% (SSC) and 42.14% (EL4). While the outputs collected from the device for larger cells were 80.38% (SSC) and 0.32% (EL4) in the control group, the percentages of cells were 21.44% (SSC) and 23.28% (EL4). Bleilevens et al. also showed in their study that this method is the best method for continuous separation of cells without labeling of red blood cells and platelets [54]. The cell separation function was slightly comparable to that of MACS and FACS devices for whole blood, for instance, purities are ranging, but as high as 99%, and throughputs are up to 48,000 cells [32, 55, 56]. Additionally, based on the data presented by Son et al. [57], at two outer wall outlets, all isolated sperm cells were obtained from the red blood cells (RBCs) and also nearly 81% of non-progressive motility sperms were successfully recovered. On the other hand, at two inner wall outlets at a flow rate of 0.52 ml min^−1^ with the system, nearly 99% of the red blood cells are successfully recovered [57]. The aforementioned devices could be made by standardized superior microfabrication systems that reduce the cost and complexity of commercialization efforts [58–62]. The device provides a new approach for cancer cell sorting with high throughput and purity.

## Conclusion

Our findings indicated that we have significantly isolated the tumor cells from spermatogonial stem cells by microfluidic chips based on cell size. The microfluidic device could be a novel tool for separation of spermatogonial cells from tumor cells. This study was the first study in this field in Iran. Future research needs to focus on a way to ensure the less chance of resuming malignant cells in an individual that has just been treated and cured of cancer.

## Data Availability

All data generated or analyzed during this study are included in this article.

## Abbreviations

SSC: Spermatogonial stem cells
DMEM: Dulbecco’s modified Eagle’s medium
CTCs: Circulating tumor cells
HSCs: Hematopoietic stem cells
CFCs: Circulating fetal cells
FACS: Fluorescence-activated cell sorting
MACS: Magnetic-activated cell sorting
