# Correction to: Separating mouse malignant cell line (EL4) from neonate spermatogonial stem cells utilizing microfluidic device in vitro

**DOI:** 10.1186/s13287-020-02081-z

**Published:** 2020-12-16

**Authors:** Behnaz Ashtari, Azar Shams, Narges Esmaeilzadeh, Sara Tanbakooei, Morteza Koruji, Mojtaba Johari Moghadam, Javad Mohajer Ansari, Adel Johari Moghadam, Ronak Shabani

**Affiliations:** 1 Shahdad Ronak Comerialization Company, Pasdaran Street, Tehran, Iran; 2 Radiation Biology Research Center, Iran University of Medical Sciences, Tehran, Iran; 3 Department of Medical Nanotechnology, Faculty of Advanced Technologies in Medicine, Iran University of Medical Sciences, Tehran, Iran; 4 Department of Anatomical Sciences, School of Medicine, Iran University of Medical Sciences, Tehran, Iran; 5 Cellular and Molecular Research Center, Iran University of Medical Sciences, Tehran, Iran; 6 School of Mechanical Engineering, Iran University of Science & Technology, Tehran, Iran; 7 Aja University of Medical Sciences, Department of Cardiology, Tehran, Iran

**Correction to: Stem Cell Research & Therapy 11, 191 (2020)**

**https://doi.org/10.1186/s13287-020-01671-1**

The original article [1] displays incorrect affiliation information; the correct affiliations for each author can be viewed in this Correction article.
